# Two-dimensional heterostructure quasi-BIC photonic crystal surface-emitting laser with low divergence

**DOI:** 10.1515/nanoph-2023-0156

**Published:** 2023-06-20

**Authors:** Renjie Tang, Yilin Shi, Hongpeng Shang, Jianghong Wu, Hui Ma, Maoliang Wei, Ye Luo, Zequn Chen, Yuting Ye, Jialing Jian, Xiaorui Zheng, Hongtao Lin, Lan Li

**Affiliations:** State Key Laboratory of Modern Optical Instrumentation, College of Information Science and Electronic Engineering, Zhejiang University, Hangzhou 310027, China; Key Laboratory of 3D Micro/Nano Fabrication and Characterization of Zhejiang Province, School of Engineering, Westlake University, Hangzhou 310030, China; Institute of Advanced Technology, Westlake Institute for Advanced Study, Hangzhou 310024, China

**Keywords:** colloidal quantum dots, low divergence, photonic crystals, quasi-BIC laser

## Abstract

High beam quality, large-area output, and small footprint are significant pursuing goals for vertical-cavity surface-emitting lasers (VCSELs), which impose strict requirements on tight light confinements with minimized radiation losses. To achieve this, bound states in the continuum (BICs) have been demonstrated as an effective way of trapping light. Here, we combine BICs and photonic bandgaps to realize a quasi-BIC single-mode photonic crystal (PhC) laser on a colloidal quantum dots (CQDs)/silicon oxide (SiO_2_) hybrid integrated platform. The PhC cavity is a defect-free hexagonal heterostructure with three regions, and the thin CQDs film is embedded within the SiO_2_ nanopillar planar array as both an optical gain material and a backbone for the PhC. The mode gaps between different regions provide the lateral confinement while the quasi-BICs near the Γ-point generate the small-divergence vertical radiation coupling, resulting in a well-defined emission concentrating within ±1.85° of the normal surface direction and an optical pumping energy density threshold of 216.75 μJ/cm^2^. Our results demonstrate the design flexibility and versatility of the quasi-BIC laser even with a low contrast of a refractive index between the PhC slab and the substrate, which has potential applications in cavity quantum electrodynamics, nonlinear optics, and integrated photonics.

## 1 Introduction

Ultra-compact lasers with high beam quality and low threshold have been a long-sought goal in the field of nanophotonics. Due to the strong electric-field confinement near the metallic particles at the subwavelength scale, plasmonic resonances are usually generated for use. However, they unavoidably suffer from high ohmic losses associated with metals [1–3]. At the wavelength scale, traditional strategies for dielectric nanolasers, such as microdisks [4–6], distributed Bragg reflectors (DBRs) [7–9], and distributed feedback (DFB) resonators [10–12], have been sufficiently demonstrated. However, microdisks have limited lasing volume and their emissions are nondirective. To trap light more effectively, it is necessary to arm DBRs and DFB resonators with larger cycle numbers, which in turn increases their footprint.

Benefiting from the controllability of photonic bandgaps (PBGs), the photonic crystal (PhC) with dimensions of the order of optical wavelengths provides an excellent platform for ultra-compact resonance cavity design. In particular, one-dimensional (1-D) PhC nanobeam [13–15] and PhC defect cavities [16, 17] feature significantly low mode volumes (*V*) close to diffraction limits (∼(*λ*/2*n*)^3^) and theoretical quality (*Q*) factors exceeding 10^7^. However, since the optical field is confined to a very small area, their emission power is typically insufficient, and divergence angles are typically large, which limits the practical applications. Recently, the concept of bound states in the continuum (BICs) has been demonstrated as a compelling design to suppress out-of-plane radiations and consequently increase the *Q* factors of planar optical resonators [18–21]. In theory, ideal BICs with infinite *Q* factors only exist in symmetric and periodic structures, which fits perfectly with the defect-free PhC cavities working at band-edge modes [22–24]. Symmetry-protected BICs at the Γ point have been mostly utilized in VCSELs due to their strong radiation suppression in the normal direction, theoretically resulting in a highly oriented VCSEL with a low lasing threshold. However, this advantage comes at the cost of limited output power.

To make BIC-PhC nanolasers more practical, two strategies for optimization have been proposed. The first one is to convert ideal BICs to quasi-BICs by breaking the symmetry or truncating the infinite size. For example, Hirose et al. [25] designed triangular-shaped air holes to break the C2 symmetry (180° rotational symmetry around the *z* axis), resulting in continuous-wave (CW) lasing with high output power (1.5 W) and high beam quality (*M* ^2^ < 1.1), while maintaining a relatively low laser threshold. The second idea involves shrinking the active region to improve the density of nanolaser arrays. Various techniques have been explored to achieve lateral confinement, such as air-suspended PhC [26], surrounding the PhC with DBRs [27, 28], and using heterostructure design [29, 30]. Considering the fabricated difficulty and actual performance, the heterostructure PhC cavities appear to be the most promising ones because they can easily form band gaps with different lattice types or by adjusting the period or duty cycle (dc). Additionally, for the slow light effects at the band edges and dispersion symmetry points in heterostructure PhCs, the interaction time between the optical field and the gain materials has been prolonged, enhancing the optical gain [29].

In this paper, we present a hexagonal heterostructure PhC cavity with a 60° rotational symmetry around the *z* axis. The laser modes operate in the mode gap formed by heterogeneous regions with different duty cycles, which slightly deviate from the Γ point and work on the state of quasi-BICs. As a result, the cavity modes can be laterally confined in the core region while maintaining the excellent characteristics of symmetry-protected BIC, with a simulated *Q* factor as high as ∼2.085 × 10^4^. To achieve large-scale laser arrays, we utilize CdSe/ZnS core–shell colloidal quantum dots (CQDs) as an optical gain material due to their ease of integration and compatibility with various substrates. The laser shows a small-angle directional emission within ±1.85° with a low threshold of 216.75 μJ/cm^2^. This provides a large-scale integrated platform for highly oriented VCSELs with high efficiency and low threshold, which greatly enhances potential applications in on-chip optical communications, sensing, and quantum information processing.

## 2 Results and discussion

**Figure 1:**
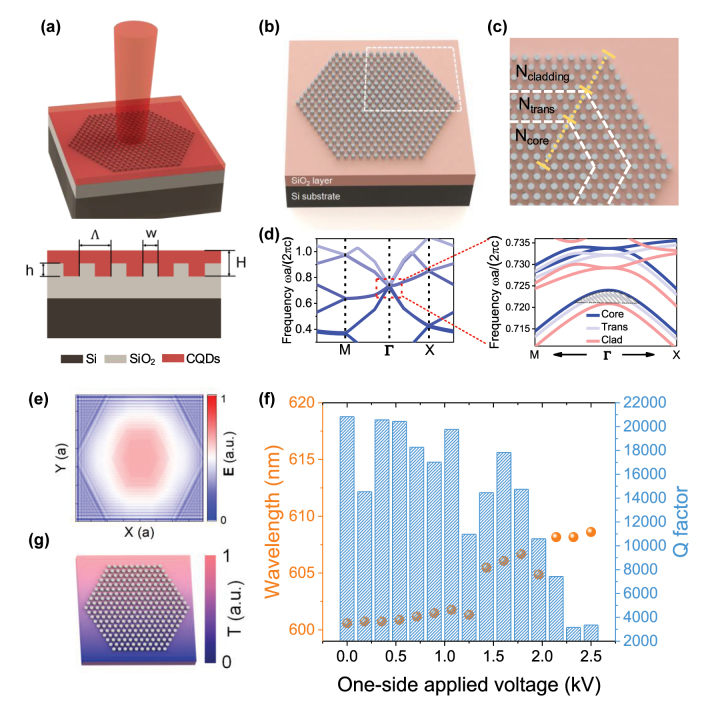
Heterostructure PhC cavity design, simulation, and calculation. (a) Schematic and longitudinal section diagrams of the heterostructure PhC laser (period Λ, nanopillar height *h*, and nanopillar width *w*), which consists of 3 layers: Si substrate, SiO_2_ (*n* ∼ 1.42), and CQDs films (*n* ∼ 1.82, thickness *H*). (b) Schematic illustration of the SiO_2_ nanopillar planar array. (c) Zoom-in view of the top right corner in (b). Different areas are circled by white dotted lines and defined as N_core_, N_trans_, and N_cladding_. (d) Left: calculated photonic band structure diagram of the PhC using the plane wave expansion (PWE) method (only shows the core region). Right: the magnified view of the red dash line framed area in left picture. Three types of regions are drawn with different color lines. (e) 3-D FDTD-simulated field distribution **E** of the fundamental resonant mode near the cavity core region. (f) A double-y plot of the FDTD-simulated *Q* factor and wavelength center as a function of one-side applied voltage. (g) A temperature distribution diagram of the device when a voltage applies to its one side in the simulation in (f).

Figure 1(a) depicts the schematic and longitudinal section diagrams of the proposed PhC laser device, consisting of a SiO_2_ pillar array (*h* = 80 nm) etched into a 3 μm thick SiO_2_ layer on a Si substrate. On top of the pillar array, the CQD film (*H* = 130 nm) is spin coated with a thickness that completely covers the SiO_2_ columns to ensure experimental reproducibility. The period of the column array and the diameter of a single cylinder are represented as Λ and *w*, and the duty cycle is the ratio of the *w* and Λ. Notably, the CQDs films serve as both the gain medium and the backbone of the PhC. Therefore, the optical properties of the CQD films play a crucial role in the device’s design (see Figure S1 of Supplementary Information). Specifically, Figure 1(b) illustrates the heterostructure of the SiO_2_ nanopillar PhC, and Figure 1(c) shows the zoom-in view of the top right corner in Figure 1(b), which is divided into three regions by white dotted lines. These regions are the core, transitional, and cladding regions arranged from the center to the edge of the structure. The duty cycles of each region (dc_core_ = 0.56, dc_trans_ = 0.54, and dc_cladding_ = 0.5) are the only parameter we use to adjust the PhC bandgap. Meanwhile, the number of columns enclosed by the yellow, blue, and pink rectangles is 20, 6, and 40, respectively. Due to the relatively smaller duty cycle, the band structure on the frequency domain of the cladding region is downward, resulting in a mode gap near the Γ-point. This gap is indicated by the shaded area in the right picture of Figure 1(d). Theoretically, the mode at the Γ-point on top of the second band is a symmetry-protected BIC (also known as a “dark mode”) for an infinite uniform hexagonal PhC lattice. In our design, however, the laser mode forms at the mode gap, where the characteristics of the dark mode are preserved. The cavity modes are vertically confined in the structure in Fano resonant states with a long lifetime, which can also be referred to as quasi-BICs. Although the designed PhC cannot support a full in-plane bandgap in the wavelength range of interest, it is expected that the coupling between the core modes in the mode gap to the higher-order cladding modes with the same frequency will be small due to the mismatch between effective indices and mode patterns. This mismatch reduces the coupling between the core and cladding modes, suppressing out-of-plane radiation and improving the laser’s overall efficiency. Additionally, the transitional region is used to prevent the mutation of the effective refractive index and minimizes the scattering loss.

We use finite-different time-domain (FDTD) to simulate the specific operating parameters of the device with a period of 435 nm. As the index contrast between the SiO_2_ and the CQDs is very low, we set a relatively high resolution of 22 grids per period, noting that the structures must mesh in the same way; otherwise, each hole in the simulation will have a slightly different size and shape. The simulated field distribution **E** of the cavity core mode in Figure 1(e) demonstrates that the cavity modes can be tightly confined laterally in the heterostructure cavity. The simulated *Q* factor of the fundamental laser mode is ∼2.085 × 10^4^ at the wavelength of 600.58 nm (Figure 1(f)). It is expected that a single-mode laser can be realized since the higher order modes in the mode gap and the band edge modes in each region are very weak. These modes can only be generated at pump power levels exceeding the material loss threshold. To investigate the sensitivity of the proposed quasi-BIC PhC to symmetry, we modeled the PhC cavity using Lumerical DEVICE. An electrode was placed on one side of the cavity, and gold was used as the heating metal. As the voltage increased, the Joule heating from the gold caused changes in the refractive index of the cavity medium. As shown in Figure 1(f), the *Q* factor remained high under a voltage of 1.3 kV, while the wavelength showed minimal variations. The results indicate that our devices operating in quasi-BIC modes are relatively insensitive to symmetry-breaking, highlighting the robustness of the proposed structure and reducing the need for highly accurate device fabrication. Figure 1(g) is the temperature distribution diagram of the device when a voltage applies to its one side in the simulation (see Figure S2 of Supplementary Information for a more detailed description of modeling methods).

**Figure 2:**
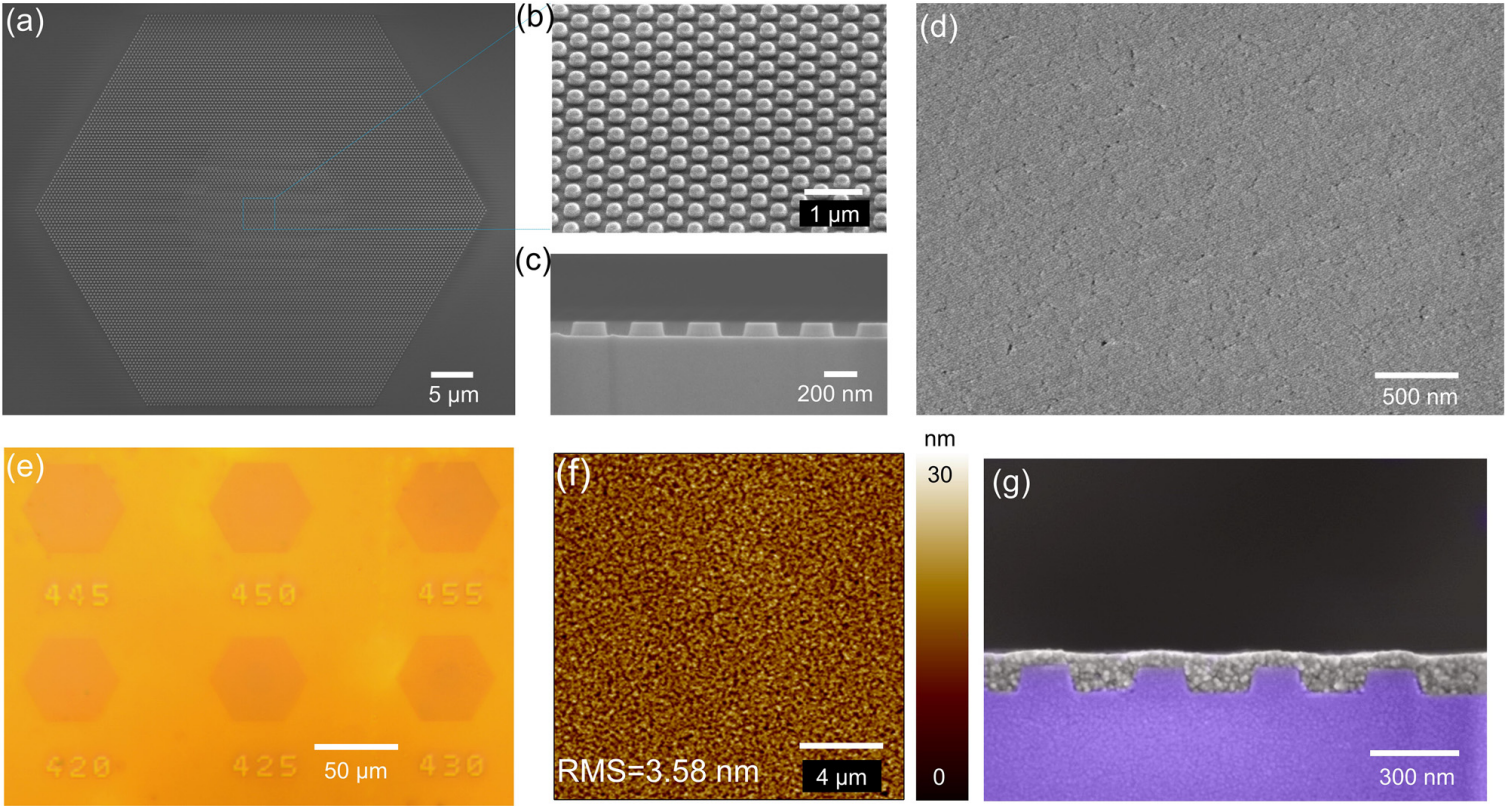
Cavity fabrication and gain material characterization. (a) Top-view SEM image of a fabricated SiO_2_ column array of a complete device without spin-coating CQDs. (b) Oblique-view SEM image of the framed area (core region) in (a) with a slant angle of 30°. (c) Longitudinal section SEM image of the core region. (d) SEM images of the CQDs film above the SiO_2_ column after spin-coating. (e) Optical microscope image of the cavity with CQDs film. (f) 20 × 20 µm^2^ AFM scanning image from the same CQDs film. The RMS surface roughness is 3.58 nm, indicating an optically smooth surface. (g) The longitudinal section SEM image of the SiO_2_ column array with the CQDs.

A 3 µm thick SiO_2_ layer was thermally grown on a silicon substrate, patterned by electron beam lithography (EBL), and then nanopillars were formed by a dry etching process (see Methods and Supplementary Information Figure S3). Scanning electron microscope (SEM) images of the fabricated SiO_2_ nanopillar planar array are shown in Figure 2(a)–(c), which exhibit few impurities and blemishes, indicating the high precision and robustness of our fabrication process. However, top edges of the pillars are not perfectly right angled, which is a major reason for the deviation between the simulated and measured results. Figure 2(d) and (e) shows the SEM and optical microscope image of the PhC templates after crosslinking of CQDs, respectively. Different PhC templates with pitches ranging from 420 nm to 465 nm (with a step of 5 nm) were fabricated, and the pitch of each cavity was etched as an identification number. Although the thickness of the CQDs film exceeds ∼54 nm above the SiO_2_ columns, the cavity can be clearly observed, indicating that the CQDs film is optically clear and spatially uniform. To further test the planarity of the film, we measured the surface roughness of the CQDs film with atomic force microscopy (AFM). The root-mean-square (RMS) surface roughness on an area of 20 × 20 µm^2^ is 3.58 nm, as shown in Figure 2(f). The CQDs with a grain size of only a few nanometers are small enough to be conformally incorporated into the nonplanar templates. Figure 2(g) shows that the actual thickness of the CQDs films is 134 ± 3 nm.

**Figure 3:**
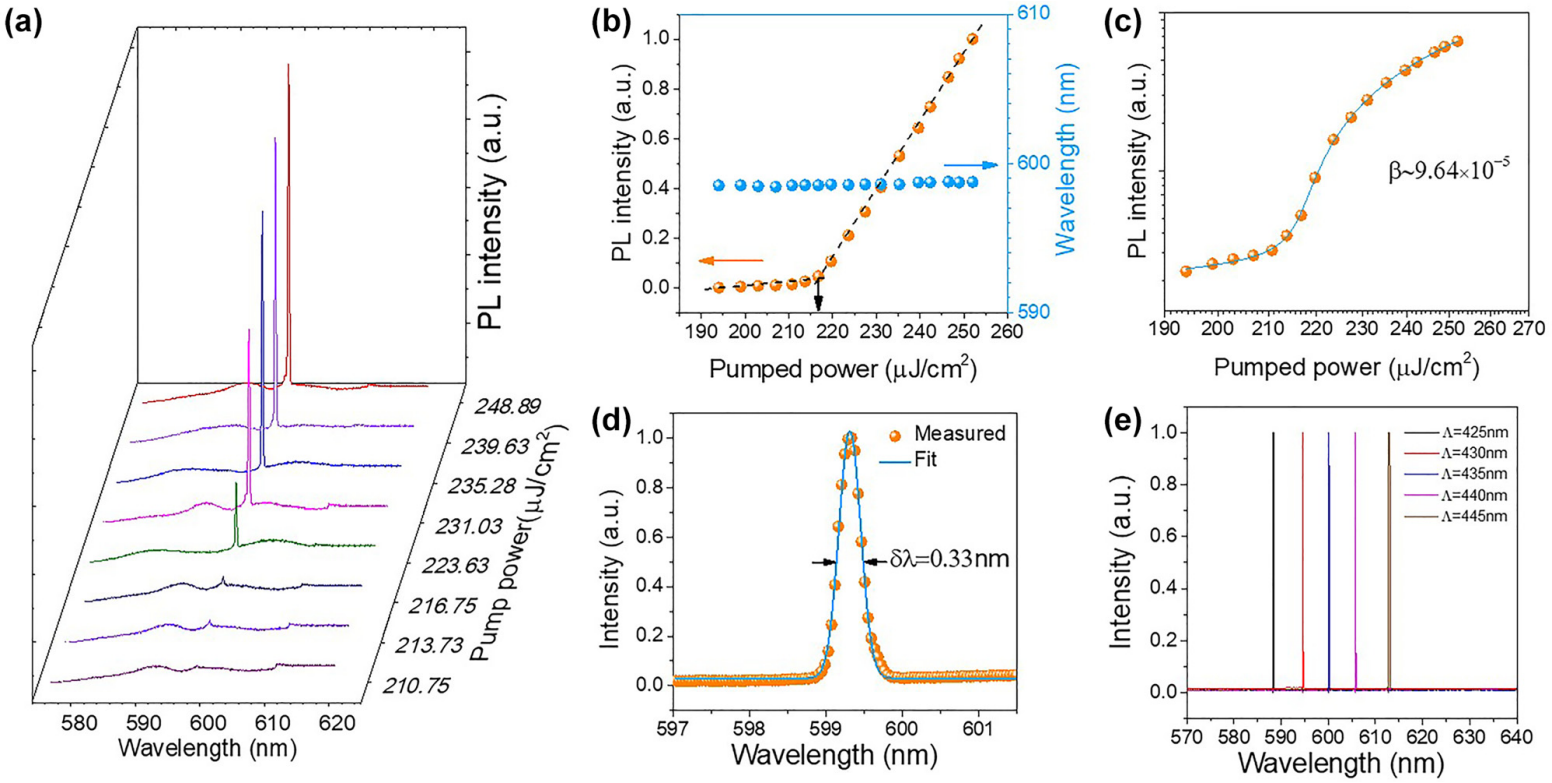
Laser properties characterization. (a) Normalized power-dependent PL spectra of a device with a pitch of 435 nm. (b) Orange dots: the L–L curve of the resonant peak intensity on a double linear scale, indicating the lasing threshold of ∼216.75 μJ/cm^2^; blue dots: power-dependent resonant wavelengths. (c) L–L curve on a double logarithmic scale with rate equation fit shown in blue solid. A spontaneous emission factor (*β*) of 9.64 × 10^−5^ is extracted. (d) *Lorentzian* fitting of the emission spectrum with a 0.33 nm FWHM for the same device. (e) Normalized PhC lasing spectra of different devices with varying pitches.

We characterized the properties of the PhC laser using our self-built femtosecond laser setup (see Methods and Supplementary Information Figure S4). Figure 3(a) depicts the normalized power-dependent photoluminescence (PL) spectra of a device with a 435 nm period, demonstrating the transition from spontaneous emission to lasing. In the light-in, light-out (L–L) curve in Figure 3(b), a sharp lasing optically pumped power turning point can be observed at 216.75 μJ/cm^2^. The experimental data of Figure 3(b) were fitted to a static rate equation model (see Supporting Information S5) on a double-logarithmic scale shown in Figure 3(c), from which a spontaneous emission factor (*β*) of 9.64 × 10^−5^ was extracted. An emission spectrum above the threshold was fitted with a *Lorentzian* function, showing a narrow full width at half maximum (FWHM) of 0.33 nm (Figure 3(d)). We believe the fitting results to be relatively accurate since at least ten pixels are located at the resonant peak. To qualitatively assess the spectral range of the CQDs gain spectrum and verify the tunability of the heterostructure PhC laser, we varied the PhC periods and studied the emission. The lasing spectra from PhC cavities with five different pitches are presented in Figure 3(e), with the single-mode lasing wavelengths ranging from 588.4 to 611.9 nm, thus covering a 23.5 nm spectral window. Notably, our substrate has minimal thermal effects within the pump power range, as evidenced by the constant center wavelength of the laser even at high pump powers (Figure 3(b)). This is in contrast to the blue shifting effects observed with quartz substrates, which necessitated a switch to sapphire in a recent study [31]. Our substrate, which is 3 µm thick and made of SiO_2_, allows for efficient heat dissipation and prevents mode field leakage, while also enabling precise EBL process steps due to its sufficient conductivity.

**Figure 4:**
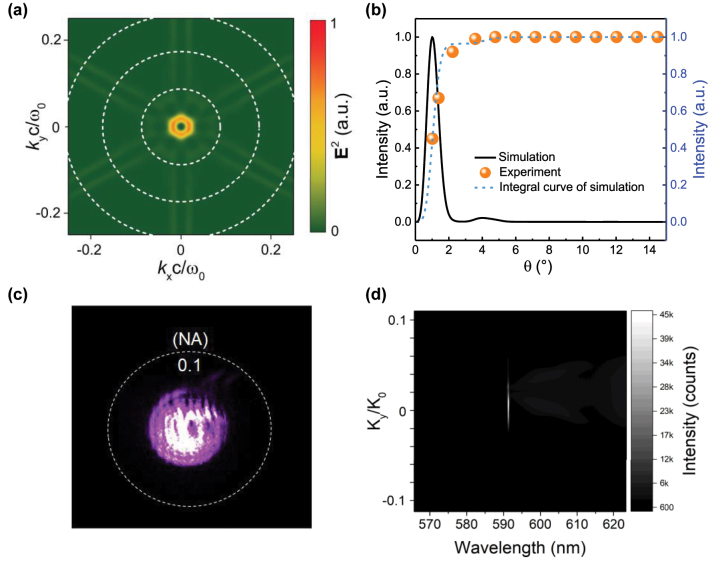
Simulation and measurement of the far field radiation. (a) Far-field radiation pattern simulated using FDTD with the dashed circles in 5° step. (b) The black line shows the simulated emission density depending on *θ*. To compare the data with the experiment, the integral curve was plotted in a blue dashed line, which represents the electric field intensity after Fourier transforms in different collection angles *θ*. The experimental data are presented as orange points. (c) Far-field radiation pattern for lasing. The dashed circle corresponds to a normalized in-plane momentum *k*_‖_/*k*_0_ of 0.1. (d) Angle-resolved PL spectra obtained from the spectrometer along *k*_*x*_ = 0.

Theoretically, since the PhC cavity is designed with the principle of the quasi-BIC near the Γ-point, vertical emission is the strongest. The far-field emission profile of the lasing mode was simulated using FDTD, as shown in Figure 4(a), which confirms that the emission is concentrated at the normal surface direction within a few degrees. We extracted the emission intensity of the lasers depending on the angle of collection (*θ*) from the far-field profile, as shown by the black line in Figure 4(b), and integrated the black line to obtain the blue dash line, which visually displays the field intensity under different collection angles. To accurately measure the divergence angle of the laser, we employed an innovative technique that utilized a thin short-pass filter containing holes of varying diameters to regulate the collection angle (*θ*) by selecting different holes in the infinity space of the setup (see Supplementary Information S6). The *θ* can be calculated using the equation *θ*(*D*) = arcsin(*D*/2*d*), where *d* = 12.5 mm represents the vertical dimension between the sample and the filter, and *D* denotes the diameter of the filter. The measured data are depicted by the orange points, which agree with the blue dashed line, indicating that the laser emission from the PhC cavity is focused within ±1.85° of the surface-normal direction. Table S1 (Supplementary Information S7) compares the divergence angles of surface-emitting nanolasers reported in recent years. To further investigate the optical properties of the symmetry-protected BIC laser, the far-field images of the laser are measured, and the angle-resolved PL spectrum is extracted from the spectrometer along *k*_*x*_ = 0 (Figure 4(c) and (d)). Our demonstrated model achieves a significantly smaller divergence angle in all directions, with a relatively simple manufacturing process and compact design, making it essential for practical applications when compared to previously reported schemes.

## 3 Conclusions

In summary, we have developed a solution-processable and highly robust SiO_2_/CQDs platform for realizing a quasi-BIC PhC laser with a low threshold of 216.75 μJ/cm^2^. Our nanostructured devices exhibit well-defined directional emission beams within ±1.85°. Moreover, the PhC laser has a flexible wavelength tunability by simply adjusting the overall pitch. The emission wavelengths of the CQDs can be tailored by changing the physical size of individual CQDs while maintaining the immobility of the composing materials, which means that the refractive index of the CQDs will not change significantly. Our device can cover a broad range across the visible and even extend to the infrared band [31, 32] within our basic design principles. As our single PhC laser cavity occupies a very small area, a larger area device such as a micro-laser array can be expected, and some interesting monochromatic or even multicolor display applications can be demonstrated [23]. Moreover, the refractive index (RI) of the SiO_2_ is close to that of most polymers, allowing for the translation of this design to a flexible substrate, which can lead to various applications such as wavelength control through mechanical stretching [33, 34] and biometric information recognition [35]. Although similar designs [36] can be realized using silicon nitride and two-dimensional van der Waals materials, stripping single-layer two-dimensional materials is difficult, and covering array devices on a large scale is challenging. Our demonstration represents a successful large-scale, large-wavelength tuning range integrated platform.

Lower thresholds and more relaxed pumping conditions, such as CW laser as a pumping source, arise from optimizing the gain material, particularly in suppressing Auger recombination losses in CQDs [37–39]. Furthermore, the high resistivity of CQD films presents a challenge for LED-like devices attempting to achieve electrically pumped lasers using traditional DFB resonators [40]; other solution-processable materials, such as hybrid perovskite, also face the same challenge [41]. With the theoretically infinite *Q* factor and diversity in design, we believe that our quasi-BIC PhC laser based on the SiO_2_/CQDs platform will have more applications with the ongoing advances in materials engineering.

## 4 Methods

### 4.1 SiO_2_ column array template fabrication

The electron beam resist ARP6200.09 was first spin-cast onto a 3 µm thick SiO_2_ layer grown by thermal oxidation on a silicon substrate. The resist was then baked at 150 °C for 60 s. Next, electron beam lithography (EBL, Raith Voyager, 50 kV) was used to generate the desired pattern, and the resist was developed to obtain the pattern. A 55 nm thick chromium layer was deposited on the patterned electron beam resist as a hard mask, followed by a lift-off process. An inductively coupled plasma (ICP, Leuven) dry etching process was then carried out to etch the substrate and form the nanopillar array. Finally, the remaining chromium hard mask was removed using chromium etchant, and the entire substrate was thoroughly cleaned with deionized water.

### 4.2 CQDs PhC laser fabrication

The CdSe/ZnS CQDs used in the experiment were obtained from Suzhou Xingshuo Nanotech Co., Ltd. The CQDs film with a thickness of approximately 122 nm was deposited onto a SiO_2_ column array using a multi-step crosslinking procedure with 1,8-diaminooctane used as a linking agent, which generated highly robust, optically clear, and spatially uniform films with a precisely controlled thickness. The procedure involved spin-casting a 25 ± 1 mg/mL CQDs octane solution to produce a film of approximately 60 nm thickness. Subsequently, a crosslinking reaction was initiated by dropwise addition of a 2.85 mg/mL 1,8-diaminooctane solution in methanol, followed by a 1 min wait. The film was then rinsed with methanol to remove any unreacted linking groups. This cycle was repeated until the desired thickness was achieved.

### 4.3 Optical characterization

Experiments were carried out at room temperature using freshly prepared CQD films on a custom-built photoluminescence setup. The pump source used was a supercontinuum femtosecond (fs) laser with a wavelength of 400 nm, a pulse width of 190 fs, and a repetition rate of 10 kHz. The pump beam was focused into a spot with a diameter of 30 µm using a 0.1 NA objective lens. The resulting PL signal was collected by the same objective lens and directed to a monochromator with a maximum grating density of 1200 g/mm. To minimize the influence of ambient light, the entire experimental system was covered with a black cloth.

## Supplementary Material

Supplementary material is available online. It includes further details on materials’ properties, modeling details of the Lumerical DEVICE, the fabrication of the SiO_2_ column array template, further details on optical characterization, rate equation analysis of L–L curves, details of divergence angle measurement, and simulation of divergence angles for traditional common PhC cavities.

## Supplementary Material

Supplementary Material Details
