# Supplementary material for: Two-dimensional heterostructure quasi-BIC photonic crystal surface-emitting laser with low divergence
Source: Nanophotonics. 2023 Jun 20;12(16):3257–65. doi: 10.1515/nanoph-2023-0156 (PMC11501963; doi:10.1515/nanoph-2023-0156)
Supplement: Supplementary file 1 — Supplementary Material Details [file j_nanoph-2023-0156_suppl_001.docx]

Supplementary Information

Two-dimensional heterostructure quasi-BIC photonic crystal surface-emitting laser with low divergence

Renjie Tang^1,2,3^, Yilin Shi^2,3^, Hongpeng Shang^2,3^, Jianghong Wu^2,3^, Hui Ma^1^, Maoliang Wei^1^, Ye Luo^2,3^, Zequn Chen^2,3^, Yuting Ye^2,3^, Jialing Jian^2,3^, Xiaorui Zheng^2,3^, Hongtao Lin^1^, Lan Li^2,3*^

^1^State Key Laboratory of Modern Optical Instrumentation, College of Information Science and Electronic Engineering, Zhejiang University, Hangzhou 310027, China

^2^Key Laboratory of 3D Micro/Nano Fabrication and Characterization of Zhejiang Province, School of Engineering, Westlake University, Hangzhou 310030, China

^3^Institute of Advanced Technology, Westlake Institute for Advanced Study, Hangzhou 310024, China

*Correspondence and requests for materials should be addressed to Lan Li. (email: lilan@westlake.edu.cn).

1. Further details on materials' properties

Figure S1: The refractive index and extinction coefficient of the CQDs film and SiO_2_ layer.

The ellipsometer (J.A. Woollam, RC2 XI+) was used to characterize the material properties, as shown in Figure S1. The refractive indexes of both CQDs and SiO_2_ exhibit smooth curves in the visible spectrum. The extinction coefficient of the CQDs is below 0.1 in the photoluminescence spectrum range, while that of SiO_2_ is zero throughout the visible light range. This indicates that the CQDs/SiO_2_ platform is an excellent choice for exploring lasing applications.

2. Modeling details of the Lumerical DEVICE


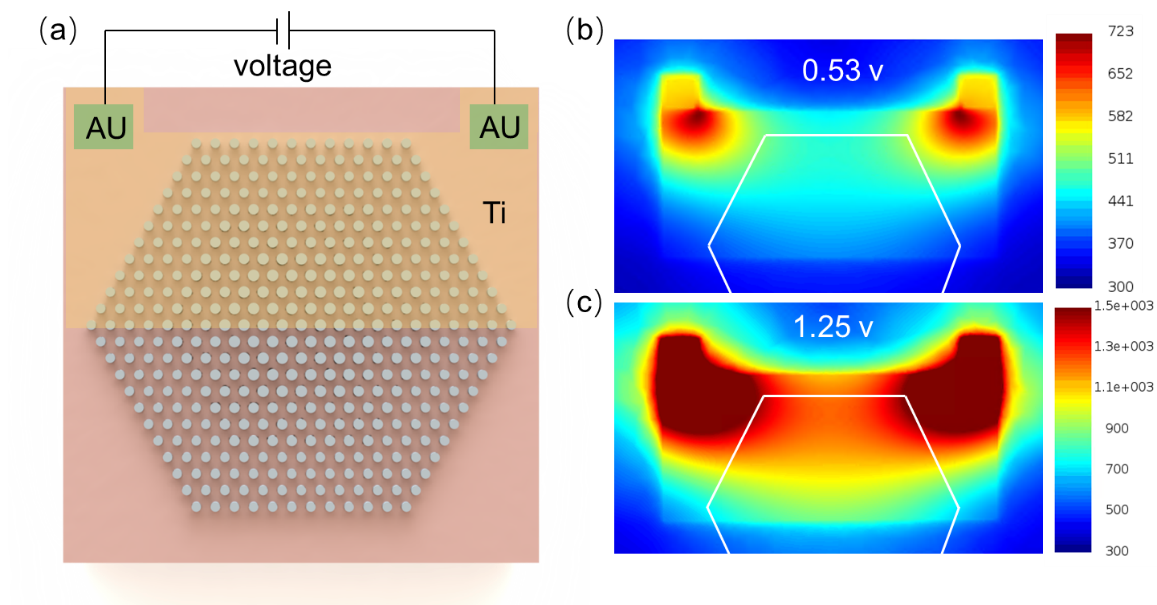


Figure S2. (a) Titanium is placed at the interface between the post and the silicon oxide layer and covers half of the array, with gold as the electrode. (b) and (c) are the temperature distribution charts when the voltage is set to 0.53 v and 1.25 v, respectively, and the unit of the color bar is Kelvin (K).

We copied the model from FDTD into the DEVICE and set a metal electrode in one section of the device. After applying voltage to the metal electrode, Joule heat will be generated, causing slight changes in the material's refractive index, ultimately leading to the breaking of the symmetry of the device. We bring the temperature distribution data under different voltages into the FDTD and re-simulate it to get Figure 1(f) in the main text. Upon analyzing the simulation results, it is apparent that the temperature reached an unexpectedly high level, which suggests that minor environmental factors have little impact on the device's performance in practical applications.

3. The fabrication of the SiO_2_ column array template

Figure S3: Electron beam lithography (EBL) based fabrication process flow of the SiO_2_ column array.

Figure S3 provides a more intuitive representation of the fabrication process flow, while specific parameters can be found in the **Method** module located in the main text.

4. Further details on optical characterization

Figure S4: The optical path figure of the test setup.

Figure S4 depicts the schematic of the PL setup utilized in device characterization. The 400nm fs laser was initially collimated by a complex collimation system and subsequently reflected into a 0.1 NA objective lens by a two-phase beam-splitter, which is transparent to beams with a wavelength exceeding 550 nm but totally reflects beams with a wavelength below 500 nm. The PL signal was collected by the same objective lens and directed to the CCD and the monochromator through a beam-splitter, respectively. A high-pass filter was placed in front of the monochromator to filter out excess pump signals and protect the monochromator.

5. Rate equation analysis of L-L curves

To model the laser emission, we employ a basic rate equation analysis for microcavity lasers[[1](#_ENREF_1), [2](#_ENREF_2)]. We found that a simplistic single-mode model with a static pump rate can effectively capture the main emission intensity characteristics. Specifically, the intensity of lasing photons (S) as a function of the pump rate (P) can be determined using a static solution, which can be expressed as follows:

$S=\frac{1}{2}(U+\sqrt{V^{2}+\frac{4\xi\left( \beta-1 \right)P}{A\tau_{s}}+\frac{4\xi(1-\xi)}{A\tau_{s}}})$ (1)

with

$U=P-1-(\frac{1}{A\tau_{s}}-1)\xi$ (2)

$V=U+\frac{2\xi}{A\tau_{s}}$ (3)

The fraction of spontaneous emission lost from the open cavity is denoted by $(1-\xi)$, where $\xi$ is the vertical guiding due to total internal reflection. The free-space spontaneous emission rate is represented by *A*, and the single exciton spontaneous emission lifetime is $\tau_{S}$. The fraction of spontaneous emission coupled into the lasing mode is represented by $\beta$. In our analysis, we neglected non-radiative recombination processes and considered only radiative decay, which implies that ${A\tau}_{S}=1$. For our PhC laser, we calculated that the transversal open-cavity losses $\left( 1-\xi\right)$ is 0.25966.

To obtain the experimental L-L curve for a certain lasing mode, we recorded the output intensity *I* as a function of pump fluence *p*. To fit the measured curve, we used the following form: *I(p)=k_3_[S(k_1_p)+k_2_p]]*, where k_1_ is the linear scaling factor that relates the excitation rate to the pump fluence, k_2_p represents the background signal that is approximately proportional to the pump flux, and k_3_ is the linear factor of the energy collected by the spectrometer to the actual output intensity. We obtained the values of k_i=1,2,3_ and $\beta$ by fitting the measured data. The fit matches the measured data well (Figure 3c in the main text), and we determined that $\beta$ is $9.64\times{10}^{-5}$.

6. Details of divergence angle measurement


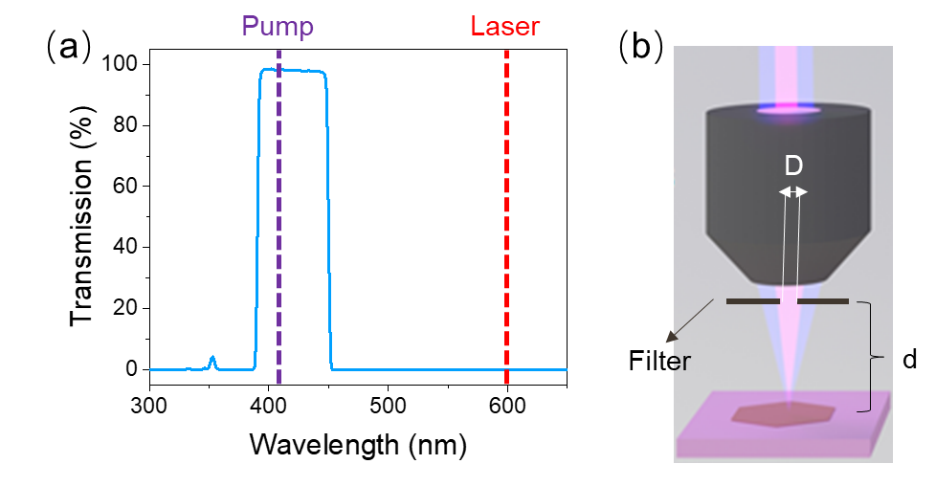


Figure S5. (a) Transmittance spectrum of the short-pass filter. (b) Schematic diagram indicating the position of the filter position. D represents the diameter of the small hole on the filter, and d represents the distance from the filter to the device surface.

We have machined small holes with different diameters on the thin short-pass filter. Because the distance from the filter to the output port of the objective lens is very close, using a regular small aperture will hinder a portion of the pump light. In the process of adjusting the size of the small hole, the energy and focusing effect of the pump light will be different, and it is impossible to control the variables. According to Figure S5, we can see that the filter is almost transparent to the pump light, so no matter how we adjust the size of the small hole, the pump light can pass through the filter completely and focus on the device's surface. However, the filter is entirely impervious to the emitted laser wavelength, so this filter is equivalent to a "small aperture" for the emitted light

7. Simulation of divergence angles for traditional common PhC cavities

|  | Schematic diagram of model | Simulated far field |
| --- | --- | --- |
| L3 point-defect PhC[[3](#_ENREF_3)] |  |  |
| Uniform hexagonal lattice PhC  (5×5 periods) |  |  |
| Uniform hexagonal lattice PhC  (10×$\times$10 periods) |  |  |
| Uniform square lattice PhC  (5×5 periods) |  |  |
| Uniform square lattice PhC  (10×$\times$10 periods) |  |  |

Figure S6: Schematic diagram of models and FDTD simulated far field of traditional common PhC cavities.

**Table S1. Comparison of micro-laser divergence angles published in recent years.**

| Laser/resonator type | Divergence angles (°) | Year | | Ref |
| --- | --- | --- | --- | --- |
| Heterostructure PhC | 1.05 (FWHM) 1.85 (1/e^2^) |  | This work | |
| PhC | 9 | 2022 | | [[4](#_ENREF_4)] |
| DBR | 3/4 (X/Y) | 2022 | | [[5](#_ENREF_5)] |
| Plasmonic-crystal | 4 | 2011 | | [[6](#_ENREF_6)] |
| PhC | 16.5/16.8 (X/Y, FWHM) | 2021 | | [[7](#_ENREF_7)] |
| Vortex bullseye | 3 | 2020 | | [[8](#_ENREF_8)] |
| DBR | 5 (FWHM) 7-8 (1/e^2^) | 2020 | | [[9](#_ENREF_9)] |
| PhC | 10 | 2019 | | [[10](#_ENREF_10)] |
| DFB | 15 | 2018 | | [[11](#_ENREF_11)] |
| DFB | 8-9 | 2016 | | [[12](#_ENREF_12)] |
| PhC+DBR | 10 | 2013 | | [[13](#_ENREF_13)] |

The L3 cavity is a widely used point-defect PhC that can achieve high Q and low modal volume (V) through the fine-tuned arrangement of six holes near the cavity edges[[3](#_ENREF_3)]. Most L3 cavity laser applications have been realized on high refractive index substrates such as Si, GaN, InGaAsP, and others. We set up the simulated model using parameters from a previous paper[[3](#_ENREF_3)], and obtained the far-field distribution of the resonant wavelength. We observed a large divergence angle and concluded that most of the output intensity was focused on the range of 50 to 60 degrees.

Furthermore, we simulate the uniform hexagonal lattice PhC, in which we select a repeat unit and use the *Bloch* boundary condition in the FDTD simulation. Since periodic arrays are finite in the actual design, we used projection functions to calculate an approximate far-field distribution. Figure S5 shows the far field distribution for the $5\times5$ and $10\times10$ periods in x and y directions, with divergence angles of 3.27°/5.62° and 1.71°/2.86° (X/Y, FWHM); 5.51°/9.55°and 2.81°/4.82° (X/Y, 1/e^2^), respectively. Similarly, we simulate the uniform square lattice PhC, and the divergence angles for the $5\times5$ and $10\times10$ periods are 4.59° and 2.29° (FWHM); 7.76°and 3.9° (1/e^2^), respectively. It is worth noting that the simulated divergence angle is a perfect state and is often smaller than the actual measured value, as shown in Table S1. Compared to previously reported schemes, our demonstrated model can realize a very small divergence angle in every direction with a relatively simple manufacturing process and a compact footprint.

References

1. Zhu Y, Xie W, Bisschop S, et al. On-chip single-mode distributed feedback colloidal quantum dot laser under nanosecond pumping. *ACS Photonics* 2017; 4: 2446-2452.

2. Stoferle T, Moll N, Wahlbrink T, et al. Ultracompact silicon/polymer laser with an absorption-insensitive nanophotonic resonator. *Nano Lett* 2010; 10: 3675-8.

3. Akahane Y, Asano T, Song B-S & Noda S. Fine-tuned high-Q photonic-crystal nanocavity. *Optics Express* 2005; 13: 1202-1214.

4. Safronov K R, Popkova A A, Markina D I, et al. Efficient Emission Outcoupling from Perovskite Lasers into Highly Directional and Long‐Propagation‐Length Bloch Surface Waves. *Laser & Photonics Reviews* 2022; 16.

5. Azad Z & Talukder M A. Simultaneously surface- and edge-emitting plasmonic laser operating in the near-infrared region. *Optics & Laser Technology* 2022; 146.

6. Huang K Y, Liu Y L, Wu C C, et al. Room-temperature two-dimensional plasmonic crystal semiconductor lasers. *Opt Express* 2021; 29: 19384-19391.

7. Chen Z, Qi A, Zhou X, et al. High Power and Narrow Vertical Divergence Laser Diodes With Photonic Crystal Structure. *IEEE Photonics Technology Letters* 2021; 33: 399-402.

8. Sun W, Liu Y, Qu G, et al. Lead halide perovskite vortex microlasers. *Nat Commun* 2020; 11: 4862.

9. Khan Z, Ledentsov N, Chorchos L, et al. Single-Mode 940 nm VCSELs With Narrow Divergence Angles and High-Power Performances for Fiber and Free-Space Optical Communications. *IEEE Access* 2020; 8: 72095-72101.

10. Wang Z, Liang Y, Meng B, et al. Large area photonic crystal quantum cascade laser with 5 W surface-emitting power. *Opt Express* 2019; 27: 22708-22716.

11. Lei C, Choquette K D, Maynard J, et al. in Vertical-Cavity Surface-Emitting Lasers XXII (2018).

12. Chang T-Y, Pan C-H, Hong K-B, et al. Quantum-Dot Surface Emitting Distributed Feedback Lasers Using Indium–Tin–Oxide as Top Claddings. *IEEE Photonics Technology Letters* 2016; 28: 1633-1636.

13. Diao Z, Bonzon C, Scalari G, et al. Continuous-wave vertically emitting photonic crystal terahertz laser. *Laser & Photonics Reviews* 2013; 7: L45-L50.
